# Latent Motivation Profiles and Doping in Sport and Exercise: An Integrative Approach Based on Achievement Goal and Self‐Determination Theories

**DOI:** 10.1111/sms.70138

**Published:** 2025-09-19

**Authors:** Ian David Boardley, Shuge Zhang, Scott Alec Gunning, James William Adie

**Affiliations:** ^1^ University of Birmingham Birmingham UK; ^2^ Hunan University of Technology Zhuzhou China; ^3^ Coventry University Coventry UK; ^4^ University of Northampton Northampton UK; ^5^ Birmingham Newman University Birmingham UK

**Keywords:** achievement goals, clean sport, doping, latent profile analysis, motivation, self‐determination

## Abstract

Utilizing adult sport (Study 1: *N* = 290; *M*_age_ = 25.0 years, SD = 10.9) and exercise (Study 2: *N* = 501; *M*_age_ = 23.9 years, SD = 7.2) samples—competing and training at a range of levels—we identified several motivational profiles, determining which profiles were most associated with doping‐related attitudes, intentions, and behaviors. Across both studies, participants responded to multi‐section questionnaires measuring goal orientations (task and ego), motivational regulations (controlled, autonomous, and amotivation), doping attitudes, doping likelihood (Study 1), doping self‐regulatory efficacy, moral disengagement, and self‐reported doping and supplement use (Study 2). Latent Profile Analysis (LPA) revealed five subgroups of motivational profiles in Study 1: Mixture Low, Task, Mixture Medium, Mixture High, and Autonomous Task. Consistent with our hypotheses, a distal outcome model of LPA revealed significant differences in doping attitudes and likelihood between profiles that suggested task goal orientation and autonomous regulation are generally linked with lower risk of doping, while ego goal orientation and controlled/amotivated regulation are linked with increased risk. In Study 2, we sought to replicate the profiles from Study 1 in a gym population and evaluate them across a wider range of doping outcomes. LPA identified four equivalent profiles to Study 1, with only the Mixture Low profile not emerging. In terms of risk for doping, distal outcome analysis supported a similar pattern of results to Study 1. Our person‐centered approach advances understanding of motivational profiles in sport and exercise, and their connection to doping risk.

## 1 Introduction

Doping, the prohibited use of image and performance‐enhancing drugs (IPEDs) (WADA, [1]), is a global problem occurring within both sport and exercise contexts [2, 3]. While prevalence estimates vary, past usage has been reported to be as high as 57% for elite athletes [4] and up to 89% among gym users [2], with sub‐elite populations evidencing the highest risk for doping [5]. With far‐reaching consequences (e.g., competition bans, reputational damage, health costs) affecting users and others [6, 7], it is unsurprising then that researchers are focused on pinpointing risk factors associated with doping to help inform effective interventions to deter use and promote clean participation [5]. Motivation is a central variable in doping research because it can help shape athletes' goals, moral functioning, and susceptibility to pressure [8, 9]. Further, understanding motivational dynamics in sport and exercise can inform more targeted and effective anti‐doping interventions. The current multi‐study paper aimed to advance the extant motivation‐doping literature by adopting a person‐centered approach integrating achievement goal [10] and self‐determination theories [11]. Specifically, we aimed to ascertain the motivational profiles of young adult sub‐elite athletes and exercisers, and which profile type(s) showed the greatest risk of undesirable attitudes, intentions, and actual use of doping. Adopting a person‐centered approach to this topic may be particularly beneficial, as the identification of at‐risk profiles has the potential to inform the development of screening tools and targeted interventions to help prevent doping.

### 1.1 Achievement Goal Theory

Achievement goal frameworks (for an overview in sport, see [12]), postulating that individuals engage in achievement contexts to demonstrate high competence—or avoid showing incompetence—have helped guide understanding of individual difference factors contributing to doping [5, 13]. According to Nicholls' [10] theory, two orthogonal goal orientations are proffered to be salient in achievement contexts, including sport and exercise. Task‐oriented individuals judge competence using self‐/task‐referenced criteria and feel most successful when demonstrating task mastery, high effort, and self‐improvement. In contrast, ego‐oriented individuals use normative criteria to evaluate competence and feel most successful when outperforming others.

Goal orientations are theoretically assumed to differentially relate to doping outcomes. For example, task‐oriented individuals are known to demonstrate high moral conduct and intrinsic motivation [12], thus, they are less likely to engage in cheating behavior (such as doping) to avoid undermining their accomplishment of self‐/task‐competence. In supporting these theoretical predictions, research has shown task orientation to hold direct and indirect associations with doping‐related outcomes [9, 14, 15], making those adopting this goal to be more protected from doping. Conversely, ego‐oriented athletes exhibit less moral conduct and hold unsportspersonlike views, both of which provide indirect evidence that ego‐oriented individuals should be more likely to use illicit methods (including doping) to achieve superiority and normative success. Consistent with this supposition, ego orientation has been associated, albeit weakly, with favorable doping attitudes and intentions [14, 15]. In contrast, however, meta‐analytical findings have inconsistently linked ego orientation and doping use [5, 13]. Despite these mixed findings, we argue that it is premature to dismiss the utility of Nicholls' goal orientation theory. For instance, there is a scarcity of research [16] that has assessed the orthogonality of goal orientation profiles across a range of doping outcomes and sport and exercise contexts. Given the centrality of orthogonality of goal orientations to achievement goal theory, this is a considerable limitation that it is important to address. In addition, there is a paucity of research that has integrated Nicholls' [10] theory with other motivational theories to explore risk factors for doping.

### 1.2 Self‐Determination Theory

One motivational theory with potential to be investigated alongside achievement goal theory when exploring doping is self‐determination theory (i.e., SDT) [17]. This theory posits that people's goal‐directed behavior in a particular domain, such as sport or exercise, is regulated by their degree of self‐determined motivation (i.e., feeling the origin of one's own choices and assimilating behaviors and values that are congruent with the self) [11]. Originally, SDT [17] focused on the distinction between intrinsic (i.e., engaging in an activity for the sake of itself), extrinsic (i.e., engaging in an activity to achieve separate outcomes, or due to internal/external pressures), and amotivation. However, contemporary SDT researchers [17, 18] operationalize motivation as *autonomous* (i.e., intrinsic and self‐determined forms of extrinsic motivation) and *controlled* (i.e., less self‐determined types of extrinsic motivation) regulations, alongside *amotivation*. SDT‐based research in the physical domain has supported theoretical predictions that higher levels of autonomous motivation are associated with optimal functioning, well‐being, and moral behaviors, whereas controlled motivation and amotivation have been linked to maladaptive outcomes including immoral functioning [19].

Similar findings have been replicated when researching doping‐related outcomes from an SDT perspective. Specifically, autonomous motivation has been linked with doping avoidance [20], and individuals with intrinsically motivated profiles have reported less proneness toward doping [21, 22]. Intrinsically (or highly autonomous) athletes are primarily concerned with deriving the inherent enjoyment from participating in sport and adopt sportspersonship values that make them unlikely to act unethically (i.e., engage in doping) [20, 23]. Despite this empirical support, some researchers [24] have reported null findings for autonomous motivation and doping variables. Thus, it is possible other motivational variables not accounted for in these studies (e.g., achievement goals) might confound some of the effects of autonomous motivation and doping variables reported to date.

The evidence concerning the associations between controlled or amotivation and doping variables, including doping use [20], has consistently supported SDT. Athletes with controlled motivational regulation, or who are amotivated, are inclined to hold more favorable attitudes toward, and be more susceptible to, doping [21, 22, 24]. For individuals with controlled or amotivation, doping may be viewed as a way of alleviating internal and external pressures (or feelings of helplessness), and increasing the chances of tangible rewards, resulting in a greater risk of doping. Importantly, combining aspects of SDT and AGT may prove to be more effective in explaining variability in doping‐related outcomes than adopting either theory in isolation.

### 1.3 An Integrated Motivational Approach

Vansteenkiste, Lens et al. [25] developed a conceptual model by integrating key tenets of AGT with SDT. More specifically, they proposed one achievement goal (i.e., task or ego) can lead to different outcomes depending on how it is regulated (i.e., autonomous vs. controlled). For example, an athlete could strive to demonstrate superior ability (ego goal) because they enjoy the challenge of striving to outperform others (autonomous reasons) or because of feelings of guilt or pressure created by significant others (controlling reasons). Likewise, a gym user could focus on mastering their weightlifting technique (task goal) out of challenge, excitement, or personal significance (autonomous reasons) or because of demanding self‐ or others' expectations (controlling reasons).

Vansteenkiste, Lens et al. [25] integrated model has been empirically supported in the sport domain, mostly in the context of sport performance, moral functioning, and well‐being [26, 27, 28]. For example, self‐determined reasons underlying task‐approach goals have been positively associated with pro‐social behavior, enjoyment, and performance satisfaction in volleyball players [27], and challenge appraisals and pride among runners [26]. Further, Vansteenkiste et al. [28] found autonomously regulated ego‐approach goals to be positively associated with indices of well‐being, whereas underlying controlling reasons for the same goal yielded a more tolerant attitude towards cheating [28]. To the best of our knowledge, no published studies have tested the combined effects of motivational regulation and achievement goals on doping in a sport or exercise context.

Before considering the application of Vansteenkiste, Lens et al. [25] integrated model for doping research, several points are noteworthy. First, most of the literature using the integrated model has assessed regulations by considering them as underlying (autonomous/controlled) reasons for pursuing a particular goal. Within this method, the goals and reasons underlying them become inextricably linked with each other, and thus, blur the interpretation of an interaction effect. A more appropriate method would be to assess motivational regulations for participation and achievement goals independently of each other [29]. Secondly, past studies have only focused on selected goals from Elliot's approach‐avoidance framework given that it would be inconceivable for a single study to examine the moderation hypothesis of motivational regulations across all six goals from Elliot's 3 × 2 model. This calls into question the issue of parsimony. Alternative goal frameworks, such as Nicholl's theory, for evaluating the integrated model have seldom been considered. An exception is a study by Gjesdal et al. [29] with student‐athletes who conducted separate conditional process models examining the indirect relationship of different goal orientations with global self‐esteem, through need satisfaction which became stronger with increasing levels of intrinsic (i.e., autonomous) regulation. The present studies considered the full spectrum of motivation (autonomous, controlled, and amotivation) and the effects of goal orientations simultaneously.

The third and final point refers to research empirically supporting Vansteenkiste, Lens et al. [25] integrated model, which has done so by adopting a variable‐centered and dimensional approach. Despite the potential of this integrated motivational model to help understand doping in sport and exercise, employing it using a variable‐centered approach may yield limited theoretical and applied insights. Specifically, it overlooks the motivational profiles of individuals who are more (or less) likely to engage in cheating/immoral behavior, such as doping. Furthermore, the nature of the constructs used (AGT and SDT) affords the possibility for an array of sub‐group combinations of orthogonal goal orientations (e.g., high/low, high all, and low all) and regulations to exist. However, the traditional variable‐centered approach negates these relationships, treating the effects of each construct as unique rather than as part of a range of coexisting variables that may influence individuals. To address this, the current study implements a person‐centered approach to identify subgroups expressing distinct motivational profiles before examining which group/s are more at risk of doping.

Few person‐centered studies exist focused on the motivational profiles of doping in sport and exercise, and none that simultaneously examine AGT and SDT constructs. Using Elliot's 2 × 2 achievement goal framework (see Elliot, [30]), Barkoukis et al. [21] examined different motivational profiles on doping, revealing that a “mastery oriented” profile (i.e., high in task approach and avoidance; low in ego‐approach and avoidance goals) was less likely to disclose self‐reported IPED use compared to athletes in groups with approach‐based profiles (high on task and ego approach goals; low on avoidance), or high achievers (high on all four types of goals). Furthermore, within the same study, Barkoukis et al. [21] conducted a separate SDT focused analysis and found that athletes belonged to three distinct profiles (intrinsic, extrinsic, and amotivated groups), with the amotivated group scoring significantly higher on past self‐reported use of doping and intentions for future use compared to the other groups. Although Barkoukis et al. [21] added to our understanding of motivational profiles for doping risk, their findings were limited to a separate profile analysis for AGT and SDT. Furthermore, the operationalization of the SDT profiles, particularly extrinsic motivation, masks varying degrees of self‐determination across its different dimensions (i.e., integrated is highly self‐determined, but external is low/more controlled). As such, we aimed to extend this person‐centered research by considering the finding that definitional aspects of construing goals are important, thus reinforcing our justification of using Nicholl's goal orientations (over approach‐avoidance goals) alongside autonomous, controlled, and amotivation regulations, providing an integrative framework to comprehensively understand the motivational profiles associated with doping.

### 1.4 The Present Research

Past SDT and AGT research has established associations between goal orientations, motivational regulations, and doping‐related outcomes. However, this work is either limited to a traditional variable‐centered approach or overlooks the combinations of motivational profiles utilizing AGT and SDT constructs when using a person‐centered approach. Thus, we tested the postulates of Vansteenkiste, Lens et al. [25] integrated model more fully by adopting a person‐centered approach, integrating goal orientations with motivational regulations. To the best of our knowledge, we are the first researchers to utilize an integrated motivational model with a person‐centered approach to study doping‐related outcomes. We examined motivation profiles for risk and protective factors for doping across two studies using both competitive athletes (i.e., Study 1) and exercisers (i.e., Study 2).

Study 1 examined whether subgroups exist among sport performers on key constructs from SDT and AGT, and whether subgroups differed in attitudes, intentions, and likelihood of doping. Study 2 then sought to evaluate whether the findings from Study 1 could be replicated with an exercise population and using alternative doping variables. For both studies, we hypothesized that a finite number of subgroups, or motivational profiles, would emerge based on sport performers' goal orientations and motivational regulations, and that the profiles would show consistency across two samples. We could not be more specific given limited person‐centered research is available to inform profile hypotheses. In hypothesizing differences across motivational profiles on doping‐related outcomes, we drew from the integrated motivational model [25] and past research [21]. It was expected that subgroups exhibiting high ego orientation with controlled/amotivation and low task orientation/autonomous regulation would be more at risk of doping, as reflected in the doping outcome variables assessed in the specific study.

## 2 Study 1—Materials and Methods

### 2.1 Participants

We recruited 290 participants (*M*_age_ = 25.02, SD = 10.86; 27.7% males) from sport teams in the UK. At the time of data collection, participants were competing at club (49.3%), university (14.8%), county (22.8%), or (inter)national level (13.1%), either in a team (62.5%) or individual sport (37.5%). The sample size fulfilled the proposed minimum requirements (i.e., ≈300 participants) for latent profile analysis [31].

### 2.2 Measures

#### 2.2.1 Achievement Motivation

The Task and Ego Orientation in Sport Questionnaire (TEOSQ), developed by Duda and Nicholls [32], was used to assess participants' task and ego orientations in sport. The questionnaire consists of 13 items, with seven items measuring task orientation (e.g., “I feel most successful in sport when I learn a new skill and it makes me want to practice more”) and six items measuring ego orientation (e.g., “I feel most successful in sport when I can do better than my friends”). Participants responded to each item on a 5‐point Likert scale ranging from 1 (*strongly disagree*) to 5 (*strongly agree*). Higher scores indicate greater task or ego orientation. The TEOSQ has demonstrated good reliability and validity in previous research [32]. Presently, Cronbach's alpha was 0.86 and 0.84 for ego and task orientation, respectively, suggesting good internal consistency. In the original scale development article, they were 0.86 and 0.89, respectively [32].

#### 2.2.2 Self‐Determined Motivation

The Sport Motivation Scale‐6, developed by Mallet et al. [33], was used to assess participants' motivation in sports. The scale consists of 24 items that measure 6 subscales of motivation, including intrinsic motivation (e.g., “For the excitement I feel when I am really involved in the activity”), four types of extrinsic motivation (e.g., External regulation– “Because it allows me to be well regarded by people that I know”), and amotivation (e.g., “I don't know anymore; I have the impression of being incapable of succeeding in this sport”). Participants responded to each item on a 7‐point Likert scale ranging from 1 (*does not correspond at all*) to 7 (*corresponds exactly*). Higher scores indicate greater levels of the respective type of motivation. The scale has demonstrated good reliability and validity in previous research [33]. For the purpose of addressing our research question, and in line with SDT studies [18], intrinsic, integrated, and identified motivation were aggregated to represent a composite score of autonomous motivation, and introjected and external regulations were used to compute a controlled motivation variable. Amotivation, the complete absence of any form of motivation, was modeled independently. Currently, Cronbach's alpha was 0.89, 0.82, and 0.88 for autonomous motivation, controlled motivation, and amotivation, respectively, suggesting very good internal consistency. In the original scale development article, they were 0.78, 0.79, and 0.86, respectively [33].

#### 2.2.3 Doping Attitudes

The Performance Enhancement Attitudes Scale (PEAS), originally developed by Petróczi and Aidman [34], was used to assess attitudes towards performance‐enhancing substances and behaviors in sports. We used the short version of the scale, developed by Nicholls, Madigan, and Levy [35], consisting of 8 items (e.g., “Using performance‐enhancing substances is an acceptable way to achieve success in sports”). Participants responded to each item on a 6‐point Likert scale ranging from 1 (*strongly disagree*) to 6 (*strongly agree*). Higher scores indicate more favorable attitudes towards the use of performance‐enhancing substances. The short version of the PEAS has demonstrated good reliability and validity in previous research [34]. Presently, Cronbach's alpha was 0.90 for doping attitudes, indicating excellent internal consistency. In the original scale development article, alpha values ranged from 0.71 to 0.91 across a range of samples [34].

#### 2.2.4 Doping Likelihood

Doping likelihood was assessed using the approach previously implemented in research [36]. This approach adopts a scenario‐based approach, asking participants to respond to hypothetical situations in which athletes might consider using performance‐enhancing substances. Participants rate the likelihood that they would engage in doping in each scenario on a 7‐point Likert scale ranging from 1 (*very unlikely*) to 7 (*very likely*). Higher scores indicate a greater likelihood of doping. Currently, the Cronbach's alpha of doping likelihood is 0.87, indicating very good internal consistency.

### 2.3 Procedures

Ethical approval was granted from Coventry University's research ethics committee, and BPS ethical guidelines were followed. Participants were recruited through the University's SONA system, sports clubs from the University and the wider community, and via social media advertisements. The study was administered via an anonymous online survey that took 10–15 min to complete. Participants were provided with instructions about the nature and requirements of the study, including their rights as participants. Following digital consent, participants responded to the psychological scales used to measure the Study 1 variables.

### 2.4 Data Analysis

We used IBM SPSS Version 28 to check missing data, descriptive statistics (i.e., mean, SD, skewness, kurtosis) of the study variables, and to prepare the study dataset for further Latent Profile Analysis (LPA) using Mplus Version 8 [37]. Following guidance [38], we first examined models that involved different numbers of profiles to determine the most representative latent motivation profile model based on the Study 1 data informed by key variables of AGT (i.e., ego orientation, task orientation) and SDT (i.e., autonomous motivation, controlled motivation, amotivation). Following Ferguson et al.'s [38] recommendations, we adopted *Log‐Likelihood* (LL), *Akaike Information Criteria* (AIC), *Bayesian Information Criteria* (BIC), and *sample‐size adjusted Bayesian Information Criteria* (SABIC) to assess model fit, used the *entropy value* to determine distinctiveness of profiles, and conducted the *Lo–Mendell Rubin Likelihood Ratio Test* (LMRT) and the *Bootstrap Likelihood Ratio Test* (BLRT) for model comparisons. With the optimal latent motivation profile model identified, we next examined the extent to which the varied motivation profiles differed in the levels of doping risk factors (e.g., doping attitudes and doping likelihood) using Bolck et al.'s [39] *BCH approach*, taking the advantage of the non‐parametric test for cross‐profile comparison. Robust maximum likelihood estimator (i.e., MLR in Mplus) was adopted to deal with potential multivariate outliers and data non‐normality in key variables of the motivation profiles [40]. We report mean and standard error (SE) scores of doping risk factors (e.g., doping attitudes, doping likelihood) for each motivation profile, with *χ* ^2^ and the corresponding *p*‐value of the comparison of each pair of profiles provided. For parsimony, we provide more replicable details of the analysis in the Supporting Information S1: Methods S1.

## 3 Study 1—Results

### 3.1 Preliminary Analyses

No missing data were found. The largest skewness was 1.38 in doping likelihood, while the largest kurtosis was 2.43 in doping attitudes. Such a distribution fulfills Kline's [41] criteria of normality requirements for structural equation modeling. The test of Mahalanobis Distance revealed five multivariate outliers (1.7%). The small number of multivariate outliers was retained for further analysis thanks to the advantage of the robust maximum likelihood estimation approach we adopted for LPA analysis [40]. No significant differences were found for gender and sport types in levels of motivation variables and doping risk factors (all *p*s > 0.05).

### 3.2 Latent Motivation Profiles

LPA found that a five‐profile model appeared representative to the Study 1 sample based on the model fit indices (i.e., LL, AIC, BIC, SABIC), entropy value, and the LMRT and BLRT test results (see Table 1 for statistical details; see Supporting Information S1: Methods S1 for model fit indices). The five‐profile model achieved the lowest LL, AIC, BIC, SABIC, and the highest entropy value compared to one‐, two‐, three‐, and four‐profile models. When increasing the number of profiles from five to six, the entropy value reduced, with the emergence of a non‐significant LMRT test and a new profile containing less than 5% of the total sample. Therefore, we retained and interpreted the five‐profile model.

**TABLE 1:** Fit indices, entropy, and model comparisons for latent profiles analysis on motivation variables across Studies 1 and 2.

Classes	LL	AIC	BIC	SABIC	Entropy	LMRT	BLRT	nC < 5%
Study 1 (athletes; *n* = 290)								
1	−2060.39	4140.78	4177.48	4145.77	—	—	—	—
2	−1983.12	3998.24	4056.95	4006.22	0.67	0.04	0.00	0
3	−1935.65	3915.30	3996.04	3926.27	0.79	0.01	0.00	0
4	−1901.30	3858.60	3961.35	3872.56	0.78	0.24	0.00	0
***5***	***−1870.03***	***3808.06***	***3932.84***	***3825.02***	***0.81***	***0.15***	***0.00***	***0***
6	−1854.80	3789.61	3936.40	3809.55	0.78	0.24	0.00	1
Study 2 (exercisers; *n* = 501)								
1	−2610.82	5241.63	5283.8	5252.06	—	—	—	—
2	−2464.46	4960.92	5028.39	4977.60	0.93	0	0	0
3	−2356.86	4757.71	4850.48	4780.65	0.83	0	0	0
***4***	***−2312.67***	***4681.35***	***4799.41***	***4710.54***	***0.84***	***0.18***	***0***	***0***
5	−2326.74	4721.49	4864.85	4756.93	0.76	0.95	0.25	1

*Note:* BLRT, *p*‐value of Bootstrap Likelihood Ratio Test; LMR, *p*‐value of Lo–Mendell Rubin test; nC < 5% = number of class membership that contains < 5% sample in that profile. The bold and italic row denotes the chosen model for further analysis.

Abbreviations: AIC, akaike information criterion; BIC, Bayesian Information Criterion; LL, Log‐Likelihood; SABIC, sample‐size adjusted Bayesian Information Criterion.

**FIGURE 1:**
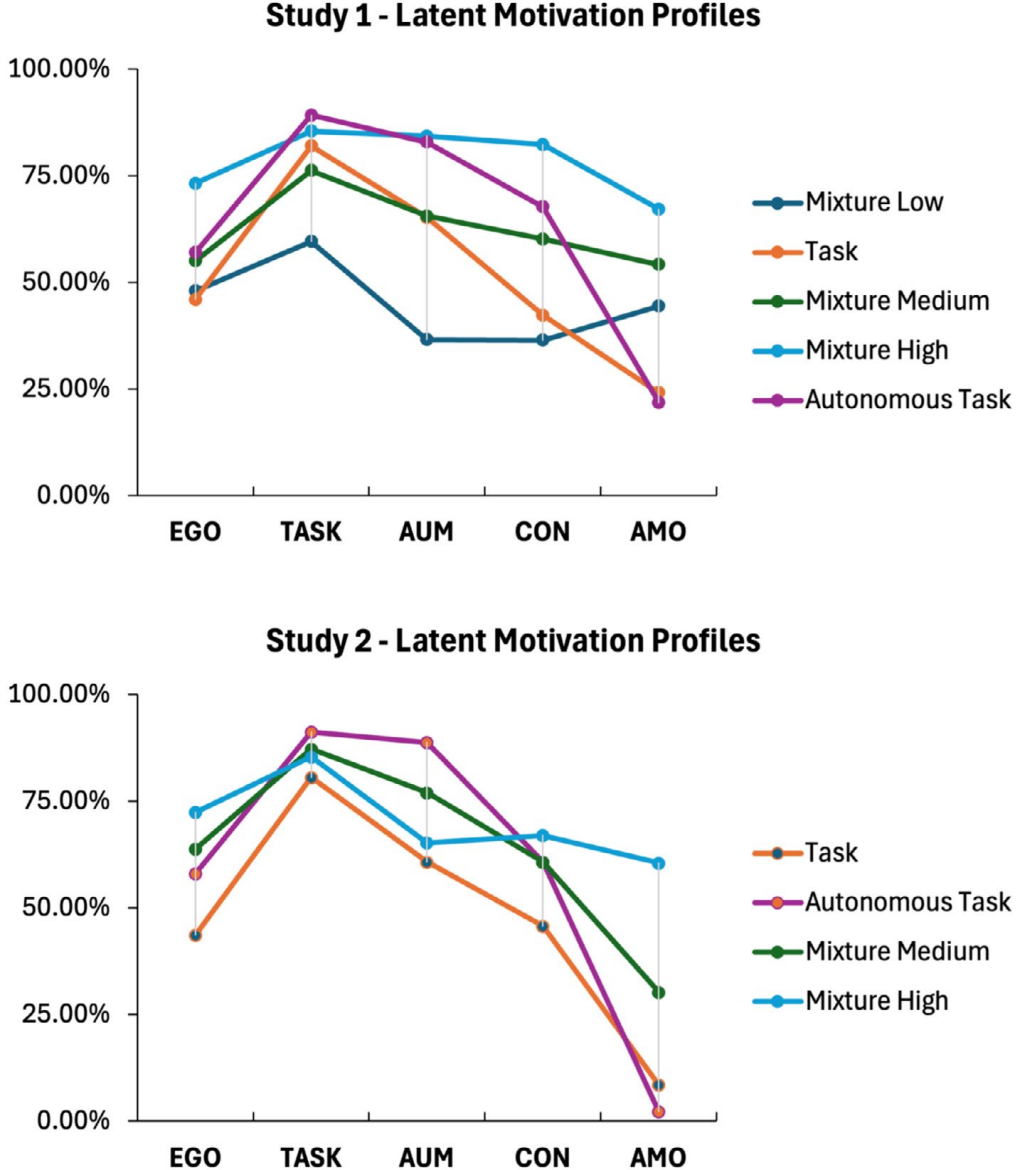
Illustration of latent motivation profiles generated in Studies 1 (athlete sample; *n* = 290) and 2 (exerciser sample; *n* = 501). The *y*‐axis denotes the percentage of mean scores in each motivation profile compared to the maximum score of the certain motivation measure thus adjusting for varied score range of different motivation measures for cross‐profiles comparison. AMO, amotivation; AUM, autonomous motivation; CON, controlled motivation; EGO, ego orientation; TASK, task orientation.

The five‐profile model supported the existence of five distinctive motivation profiles. It consisted of a *mixture low profile* (5.51%) that observed relatively low scores for all five motivation variables, a *task profile* (33.45%) that observed high task orientation scores but relatively low or medium‐range scores for the other four motivation variables, a *mixture medium profile* (21.03%) that observed medium or medium‐high scores for all five motivation variables, a *mixture high profile* (5.52%) that observed relatively high scores for all five motivation variables, and a *task autonomous profile* (34.48%) that observed simultaneous high task orientation and autonomous motivation with medium ego orientation, controlled motivation, and low amotivation. Table 2 displays the detailed descriptives of the five motivation profiles, with an illustration of the profiles provided in Figure 1 (top).

**TABLE 2:** Descriptives of the chosen latent profile models in Studies 1 and 2.

Profiles	% of sample	Scores	EGO	TASK	AUM	CON	AMO
Study 1 (athletes; *n* = 290)							
1‐Mixture low	5.51	Mean	2.40	2.98	2.56	2.55	3.11
		*% max*	*48.00*	*59.60*	*36.57*	*36.43*	*44.43*
2‐Task	33.45	Mean	2.30	4.10	4.56	2.96	1.69
		*% max*	*46.00*	*82.00*	*65.14*	*42.29*	*24.14*
3‐Mixture medium	21.03	Mean	2.75	3.81	4.59	4.21	3.79
		*% max*	*55.00*	*76.20*	*65.57*	*60.14*	*54.14*
4‐Mixture high	5.52	Mean	3.66	4.27	5.90	5.76	4.70
		*% max*	*73.20*	*85.40*	*84.29*	*82.29*	*67.14*
5‐Autonomous task	34.48	Mean	2.85	4.46	5.80	4.74	1.53
		*% max*	*57.00*	*89.20*	*82.86*	*67.71*	*21.86*
Study 2 (exercisers; *n* = 501)							
1‐Task	20.96	Mean	2.18	4.03	2.43	1.83	0.34
		*% max*	*43.60*	*80.60*	*60.75*	*45.75*	*8.50*
2‐Mixture medium	19.08	Mean	3.19	4.36	3.08	2.43	1.21
		*% max*	*63.80*	*87.20*	*77.00*	*60.75*	*30.25*
3‐Mixture high	9.50	Mean	3.62	4.27	2.61	2.68	2.42
		*% max*	*72.40*	*85.40*	*65.25*	*67.00*	*60.50*
4‐Autonomous task	54.60	Mean	2.90	4.56	3.55	2.43	0.09
		*% max*	*58.00*	*91.20*	*88.75*	*60.75*	*2.25*

*Note:* The italicised values are not tested for significance. % of sample = percentage of the participants in a certain profile compared to the total sample size; mean = average score of a certain profile; % max = percentage of the profile mean score compared to the maximum possible score (thus adjusting the different ranges of varied motivation measures for comparison within and between profiles).

Abbreviations: AMO, amotivation; AUM, autonomous motivation; CON, controlled motivation; EGO, ego orientation; TASK, task orientation.

### 3.3 Relative Risk for Doping Across Motivation Profiles

When assessing the relative risk for doping across the five motivation profiles, the results were consistent in that *autonomous task*, *task*, and *mixture low* profiles had the three lowest risk profiles, respectively, for both doping attitudes and doping likelihood. Significantly greater pro‐doping attitudes were found for the *mixture medium profile* in comparison to the *task* (*χ* ^2^ = 20.96, *p* ≤ 0.001) and *autonomous task* (*χ* ^2^ = 34.14, *p* ≤ 0.001) profiles, and for the *mixture high profile* in comparison to the *task* (*χ* ^2^ = 7.75, *p ≤ 0.001*) and *autonomous task* (*χ* ^2^ = 9.16, *p* ≤ 0.001) profiles. Finally, the *mixture medium profile* had greater doping likelihood in comparison to the *task* (*χ* ^2^ = 8.81, *p* ≤ 0.001) and *autonomous task* (*χ* ^2^ = 18.16, *p* ≤ 0.001) profiles. No other significant results were found in cross‐profile comparisons (see Table 3 for all statistical details).

**TABLE 3:** Study 1 cross‐profiles comparison on doping attitude and doping likelihood (athletes; *n* = 290).

Profiles	Doping attitude		Doping likelihood	
	Mean	SE	Mean	SE
1‐Mixture Low	2.26	0.23	2.38	0.53
2‐Task	2.00	0.07	1.84	0.13
3‐Mixture Medium	2.71	0.13	2.64	0.22
4‐Mixture High	3.20	0.43	2.53	0.52
5‐Autonomous Task	1.87	0.06	1.56	0.12

	*χ* ^2^	*p*	*χ* ^2^	*p*
Profile 1 vs. 2	1.15	0.28	0.96	0.33
Profile 1 vs. 3	2.77	0.10	0.20	0.66
Profile 1 vs. 4	3.73	0.05	0.04	0.84
Profile 1 vs. 5	2.70	0.10	2.32	0.13
Profile 2 vs. 3	20.96	0.00	8.81	0.00
Profile 2 vs. 4	7.75	0.01	1.70	0.19
Profile 2 vs. 5	1.57	0.21	2.31	0.13
Profile 3 vs. 4	1.15	0.28	0.04	0.85
Profile 3 vs. 5	34.14	0.00	18.16	0.00
Profile 4 vs. 5	9.16	0.00	3.25	0.07

*Note:* Higher scores in doping attitude and doping likelihood indicate greater risk for doping. *χ* ^2^ = chi‐square statistics in the BCH's approach for cross‐profile comparison on distal outcomes.

Abbreviation: SE, standard error.

## 4 Study 1—Discussion

In line with the well‐documented benefits of task orientation and autonomous motivation as protective factors for doping, Study 1 suggested lower pro‐doping attitude and doping likelihood in motivation profiles consisting of high task orientation or simultaneous high task orientation and autonomous motivation, especially when comparing to the *Mixture Medium* and *Mixture‐High* profiles. However, although simulation studies of latent profile analysis suggest ≈300 participants should have been adequate, ideally a sample of at least 500 participants should be recruited in studies involving latent profile analysis [31]. The Study 1 sample, therefore, may have been underpowered to detect an optimal profile solution. Additionally, the detected profile could be specific to the particular domain, or even the sample. As such, it was important to assess whether the profile could be replicated with a separate sample from an alternative physical domain (i.e., exercise). This was especially so given the unexpected emergence of a *Mixture Low* profile (i.e., low in all motivation variables). When testing for replicability in an alternative physical domain, it would also be useful to examine alternative risk factors for doping, as well as protective factors. These issues were addressed through Study 2.

## 5 Study 2—Materials and Methods

### 5.1 Study Design

To address the limitations of Study 1 and to examine the generalizability of the Study 1 findings, we sought to replicate our findings with a larger sample recruited from gym settings. The benefits of recruiting regular exercisers from gym settings were twofold. First, it enabled us to examine the extent to which the motivation profiles that emerged from the sporting contexts were replicable in exercise settings. Second, we would be more confident about the active level of the participants involved in the study and thus could examine if the emergence of a *Mixture Low* profile was genuine. We expected a largely consistent motivation profile solution to the Study 1 findings, except the mixture low profile, which we did not expect to emerge within the active population we planned to sample from in Study 2. We also implemented additional measures for protective and risk factors for doping to understand a fuller picture of the relative risk of varied motivation profiles regarding doping.

### 5.2 Participants

We recruited 501 regular (i.e., train ≥ 1/week for a minimum of 6 months) gym participants (*M*_age_ = 23.89 years, SD = 7.24; 58% males) in the UK. On average, these participants attended their gym 3.46 times (SD = 1.44) for 1.31 h (SD = 0.48) per week. At the time of data collection, the majority attended mainstream gyms (94.6%) and engaged in a combination of strength and cardio training (53.9%), while the rest attended hardcore^1^ gyms (5.4%) and participated in strength (33.7%) or cardio (12.4%) training only. Approximately three‐quarters (i.e., 76.0%) of the sample played sport as well as attending the gym. Of these, 66.4% played a team sport. The sample size was desirable in balancing between model fit and profile accuracy [31], and a further increase in sample size may not have led to a more desirable model fit and could have resulted in less meaningful or difficult‐to‐interpret profiles [42].

### 5.3 Measures

#### 5.3.1 Achievement Motivation

The Goal Orientation in Exercise Measure [43], was used to assess participants' task and ego orientations in exercise. The questionnaire consists of 10 items, with five items measuring task orientation (e.g., “I feel satisfied when I master a new exercise skill.”) and five items measuring ego orientation (e.g., “I enjoy exercising when I am the best in the group.”). Participants responded to each item on a 7‐point Likert scale ranging from 1 (*strongly disagree*) to 7 (*strongly agree*). Higher scores indicate greater task or ego orientation. The scale has demonstrated acceptable to good reliability and validity in previous research, with values of 0.78 and 0.88, respectively, for task and ego goal orientations [43]. Presently, Cronbach's alpha was 0.89 and 0.90 for ego and task orientation, respectively, suggesting very good to excellent internal consistency.

#### 5.3.2 Self‐Determined Motivation

The Behavioral Regulation in Exercise Questionnaire‐3 [44, 45] was used to assess participants' motivation regulation in exercise. The scale consists of 25 items that measure different types of motivation, including intrinsic motivation (e.g., “I exercise because it's fun.”), external regulation (e.g., “I exercise because other people say I should.”), and amotivation (e.g., “I don't see why I should have to exercise.”). Participants responded to each item on a 5‐point Likert scale ranging from 0 (*Not true for me*) to 4 (*Very true for me*). Higher scores indicate greater levels of the respective type of motivation. Currently, Cronbach's alpha was 0.73, 0.75, and 0.74 for autonomous motivation, controlled motivation, and amotivation, respectively, suggesting good internal consistency. The scale demonstrated good reliability and validity in the original scale development work, with alpha values ranging from 0.73–0.86 and 0.78–0.92 in the two relevant validation papers [44, 45].

#### 5.3.3 Doping Attitudes

The multidimensional doping attitudes scale [46] was used to measure functional and moral doping attitudes. Eight items (e.g., “doping will make my results better”) were used to measure functional attitudes and six items (“doping is morally wrong”) for moral attitudes. Participants were asked to report their level of agreement using a six‐point Likert scale from 1 (*strongly disagree*) to 6 (*strongly agree*). Cronbach's alpha was 0.74 and 0.76, respectively, for functional and moral doping attitudes, indicating acceptable internal consistency.

#### 5.3.4 Doping Self‐Regulatory Efficacy

The Doping Self‐Regulatory Efficacy Scale was used to measure an individual's confidence in their ability to resist personal and social pressures to engage in doping (i.e., doping self‐regulatory efficacy; [47]). The scale consists of six items (e.g., “…resist doping even if your training group encouraged you to do it?”), and participants respond following a prompt (i.e., “How confident are you right now in your ability to…”) using a 5‐point scale ranging from 1 (*No Confidence*) to 5 (*Complete Confidence*). The scale has been shown to produce reliable and valid scores, with excellent levels of internal consistency in both samples (i.e., Sample 1 = 0.93; Sample 2 = 0.94) from the original validation paper [47]. Presently, Cronbach's alpha was 0.92, indicating excellent internal consistency.

#### 5.3.5 Doping Moral Disengagement

The Doping Moral Disengagement Scale—Short was used to measure an individual's propensity to rationalize and justify doping using a series of psychosocial mechanisms (i.e., doping moral disengagement; [47]). The scale consists of six items (e.g., “Risks associated with doping are exaggerated”), and participants rate their level of agreement with each item using a 7‐point scale ranging from 1 (*Strongly Disagree*) to 7 (*Strongly Agree*). The scale has been shown to produce reliable and valid scores with good levels of internal consistency shown in both samples (i.e., Sample 1 alpha = 0.86; Sample 2 alpha = 0.89) from the original validation paper [47]. Currently, Cronbach's alpha is 0.76, indicating acceptable internal consistency.

#### 5.3.6 Performance Supplement and Prohibited Substance Use

Frequency of performance supplement and prohibited substance use was assessed using the approach of Boardley et al. [48]. Using this approach, we presented six performance supplements (e.g., creatine, protein, caffeine) and 28 prohibited image and performance‐enhancing substances (e.g., anabolic steroids, masteron, testosterone, trenbolone) and asked participants to record all substances and methods they have used in the past month for performance enhancement or enhanced recovery. For those used, participants then report frequency of use (i.e., less than once per week, weekly, or 3 or more times per week). Each substance used is multiplied by the frequency of use, and the resulting values are summed up to provide the value used in data analysis. This approach has been used successfully in past research [48, 49].

### 5.4 Procedures

Recruitment for the project began after obtaining ethical approval from the ethics committee of the lead author's institution. Participants were recruited through gym managers. We sought permission from these managers to invite gym members to participate in the study. Once permission was granted, potential participants were approached in the gym reception area and invited to take part. Prior to completing the questionnaire, all participants were informed about the overarching aims of the study and the rights of participants. The importance of honest responses was emphasized, and potential participants were assured their responses would remain confidential and be used solely for research purposes. Participants provided informed consent before completing the study questionnaire using a pen‐and‐paper questionnaire, which took approximately 10–15 min.

### 5.5 Data Analysis

Data analytical strategies were identical to Study 1.

## 6 Study 2—Results

### 6.1 Preliminary Analyses

No missing data were found. The largest skewness (−1.83) and kurtosis (3.57) values were observed in doping self‐regulatory efficacy, which fulfilled Kline's [41] normality requirement for LPA. The test of Mahalanobis Distance revealed eight multivariate outliers (1.6%) which were retained thanks to the advantage of the robust maximum likelihood approach adopted for LPA. Compared to females, male gym participants reported higher functional doping attitudes (*M*_difference_ = 0.39, *p* ≤ 0.001; 95% CI [0.19, 0.59]) and more frequent performance supplement use (*M*_difference_ = 1.47, *p* ≤ 0.001; 95% CI [0.90, 2.04]). However, no gender difference was found in the variables of AGT (i.e., ego orientation, task orientation) and SDT (i.e., autonomous motivation, controlled motivation, amotivation) (all *p*s > 0.05). Those attending gyms for strength training only reported greater functional doping attitudes than those attending for cardio training (*M*_difference_ = 0.56, *p* ≤ 0.001; 95% CI [0.15, 0.96]) but not those who attended gyms for both strength and cardio training. Participants attending gyms for cardio training only reported less frequent performance supplement use than strength‐only (*M*_difference_ = −3.35, *p* ≤ 0.001; 95% CI [−4.45, −2.24]) and mixed training participants (*M*_difference_ = −1.77, *p* ≤ 0.001; 95% CI [−2.82, −0.72]).

### 6.2 Latent Motivation Profiles

LPA found that a four‐profile model represented the Study 2 sample based on model fit and profile accuracy criteria (see Table 1 for details). Compared to one‐, two‐, and three‐profile models, the four‐profile model yielded the smallest LL, AIC, BIC, and SABIC (i.e., superior model fit), an entropy value of 0.84 (i.e., desirable profile distinctiveness), and consistent non‐significant BLRT tests (i.e., better model fit). When increasing the number of profiles from four to five, the latent profile model fits became poorer, and the BLRT test turned significant, whilst the additional profile only contained less than 5% of the study sample.

More specifically, the four‐profile model replicated the model found in Study 1 except for the *mixture low profile*, which was absent. The four distinctive profiles (i.e., *task*, *mixture medium*, *mixture high*, and *autonomous task*) shared identical features as those that emerged from the Study 1 sample. The prevalence of each motivation profile from Study 2 was similar to that of Study 1; the *autonomous task* profile (54.60%) was most represented in Study 2, followed by the *task profile* (20.96%), the *mixture medium profile* (19.08%), and the *mixture high profile* (9.50%). Further examination of gender differences revealed that the prevalence of the four distinctive motivational profiles did not differ between female and male participants (all *p*s > 0.05), suggesting homogeneity of motivation profiles by gender. Table 2 displays the detailed descriptives of the four motivation profiles, with an illustration of the profiles provided in Figure 1 (bottom).

### 6.3 Relative Risk for Doping Across Motivation Profiles

When assessing relative doping attitudes across the four motivation profiles, the results show considerable consistency (see Table 4 for statistical details). Specifically, the *task profile* was the lowest in functional attitudes (i.e., lowest risk for doping), followed by the *autonomous task profile*, the *mixture medium profile*, and the *mixture high profile*. The *task profile* demonstrated significantly lower functional attitudes than the *autonomous task* (*χ* ^2^ = 17.45, *p* ≤ 0.001), *mixture medium* (*χ* ^2^ = 16.03, *p* ≤ 0.001), and *mixture high* (*χ* ^2^ = 20.79, *p* ≤ 0.001) profiles. No other significant cross‐profile differences were found for functional doping attitudes. When examining moral attitudes, the autonomous task profile had the strongest attitudes (i.e., lowest risk for doping), followed by the task profile, the mixture medium profile, and the mixture high profile. The *autonomous task profile* demonstrated significantly higher moral attitudes than the *task* (*χ* ^2^ = 13.92, *p* ≤ 0.001), *mixture medium* (*χ* ^2^ = 7.67, *p* ≤ 0.001), and *mixture high* profiles (*χ* ^2^ = 42.13, *p* ≤ 0.001). The mixture high profile also demonstrated significantly lower moral attitudes compared to the task (*χ* ^2^ = 10.06, *p* ≤ 0.001) and mixture medium profiles (*χ* ^2^ = 12.79, *p* ≤ 0.001). No other significant cross‐profile differences were found for moral doping attitudes.

**TABLE 4:** Study 2 cross‐profiles comparison on functional attitude, moral attitude, doping self‐regulatory efficacy, doping moral disengagement, and frequency of performance substances and prohibited substances use (exercisers; *n* = 501).

Profiles	Functional attitude		Moral attitude		Doping self‐regulatory efficacy		Doping moral disengagement		Performance substances use		Prohibited substances use	
	Mean	SE	Mean	SE	Mean	SE	Mean	SE	Mean	SE	Mean	SE
1‐Task	3.26	0.15	3.78	0.27	4.49	0.08	2.47	0.11	1.35	0.32	0.39	0.11
2‐Mixture Medium	4.14	0.11	4.06	0.28	4.49	0.08	3.00	0.12	2.43	0.40	0.16	0.10
3‐Mixture High	4.23	0.20	2.28	0.39	4.43	0.10	3.06	0.22	2.19	0.43	0.83	0.38
4‐Autonomous Task	3.97	0.07	4.91	0.11	4.48	0.06	2.93	0.07	3.68	0.23	0.39	0.10

	*χ* ^2^	*p*	*χ* ^2^	*p*	*χ* ^2^	*p*	*χ* ^2^	*p*	*χ* ^2^	*p*	*χ* ^2^	*p*
Profile 1 vs. 2	20.79	0.00	0.50	0.48	0.00	0.99	10.03	0.00	4.20	0.04	2.24	0.13
Profile 1 vs. 3	16.03	0.00	10.06	0.00	1.40	0.24	5.83	0.02	2.51	0.11	1.28	0.26
Profile 1 vs. 4	17.45	0.00	13.92	0.00	0.01	0.91	11.45	0.00	31.69	0.00	0.00	0.96
Profile 2 vs. 3	0.16	0.69	12.79	0.00	1.29	0.26	0.06	0.81	0.16	0.69	2.78	0.10
Profile 2 vs. 4	1.45	0.23	7.67	0.01	0.01	0.91	0.20	0.66	7.25	0.01	2.86	0.10
Profile 3 vs. 4	1.56	0.21	42.13	0.00	1.47	0.23	0.29	0.59	9.52	0.00	1.26	0.26

*Note:* Higher scores in functional attitude and doping moral disengagement, lower scores in moral attitude and doping self‐regulatory efficacy indicate greater risk for doping. *χ* ^2^ = chi‐square statistics in the BCH's approach for cross‐profile comparison on certain distal outcomes.

Abbreviation: SE, standard error.

When examining cross‐profile differences in doping moral disengagement, similar findings emerged. The *task profile* was the lowest (i.e., lowest risk for doping), followed by the *autonomous task profile*, the *mixture medium profile*, and the *mixture high profile*. The *task profile* demonstrated significantly lower doping moral disengagement than the *autonomous task* (*χ* ^2^ = 11.45, *p* ≤ 0.001), *mixture medium* (*χ* ^2^ = 10.03, *p* ≤ 0.001), and *mixture high* profiles (*χ* ^2^ = 5.83, *p* = 0.02). No other significant cross‐profile differences were found for doping moral disengagement. In contrast, all motivation profiles appeared similar in levels of doping self‐regulatory efficacy (all *p*s > 0.05; see Table 4).

No significant cross‐profile differences were detected for prohibited substance use (all *p*s > 0.05; see Table 4). In contrast, for performance supplement use, we found the *autonomous task* profile had the highest levels of use, followed by the two *mixture profiles* and the *task profile* (i.e., lowest use). The *autonomous task profile* demonstrated significantly more frequent performance supplement use than the *task* (*χ* ^2^ = 31.69, *p* = 0.00), *mixture medium* (*χ* ^2^ = 7.25, *p* = 0.00), and *mixture high* profiles (*χ* ^2^ = 9.52, *p* = 0.00). No other significant cross‐profile differences were found for supplement use (see Table 4 for more details).

## 7 General Discussion

Through two studies, we aimed to evaluate Vansteenkiste, Lens et al. [25] integrated model by using a person‐centered approach, examining motivation profiles combining goal orientations and motivational regulations for various risk and protective factors for doping in competitive athletes and exercisers. Moving beyond the previous variable‐centered approaches that have been dominant within the doping literature, through the use of a person‐centered approach we were able to identify for the first time four motivational profiles that showed differences across a range of risk and protective factors for doping. Furthermore, we were able to show these profiles were consistent across two separate samples, as was the relative risk for doping across the profiles, even when different doping factors were examined. As such, the current study makes important contributions to knowledge in three primary areas. First, our research provides support for Vansteenkiste, Lens et al.'s [25] integrated model, adding to our understanding of the motivational profiles that exist in sport/exercise populations. Next, this research contributes significant understanding of motivational profiles that may be at greatest risk for doping. Finally, from a methodological standpoint, our work demonstrates the benefits of research that adopts a person‐centered approach when examining motivation and/or doping. In the discussion that follows, we consider the nature of the motivational profiles identified, what may explain the differences observed in doping risk/protection, and implications for our understanding of doping and its application in applied practice.

Four motivational profiles—*Autonomous‐Task, Task, Mixture‐Medium*, and *Mixture‐High*—were represented in the data from both studies. In both samples, *Autonomous‐Task* was the most common motivational profile (34.5% Study 1; 54.6% Study 2). This profile consisted of those who scored higher on task orientation and autonomous motivation than on other goal orientations or motivational regulations. This profile reflects individuals who feel competent in sport or exercise when they achieve task‐related goals (e.g., mastery of a skill), judge their progress using self‐referenced criteria, and engage in sport or exercise out of genuine interest and/or personal value, underpinned by a sense of volition and choice. The prevalence of similar motivational profiles characterized by high task orientation and self‐determined motivation is evident in other populations, such as student‐athletes [50]. The *Task* profile was the second most common in both samples (33.0% Study 1; 20.9% Study 2), being characterized by higher scores on task goal orientation than on ego goal orientation and the three motivational regulations. Like *Autonomous‐Task*, this profile represents feeling competent due to task mastery based on self‐referenced criteria. In contrast, though, this profile is not represented by a dominant form of motivational regulation, with both autonomous and controlled regulation lower than for *Autonomous‐Task*. In general, their reasons for participating in sport/exercise are weaker for this profile compared to *Autonomous‐Task*. The profile is consistent with research by McNeil and Wang [51], who found a “high‐task mastery” cluster to make up 19% of their elite youth soccer sample.

The *Mixture‐Medium* profile was the third most common profile in both studies (i.e., 21.0% Study 1; 19.0% Study 2). Unlike the previous profiles, which were characterized by at least one relatively high score on a motivational construct, the *Mixture‐Medium* profile represented a profile in which scores were moderate and mostly consistent across task and ego orientations and controlled and autonomous regulations. This profile reflects individuals who judge competence using both self‐ and other‐referenced criteria and participate in sport/exercise for both autonomous and controlled reasons. This profile is consistent with person‐centered motivation research by Zuber et al. [52] who found that an “average motivated players” cluster represented 21.1% of their youth football sample. The cluster included average goal orientations and self‐determination scores, and group membership was found to remain stable across two football seasons. Of the four profiles found in both samples, *Mixture‐High* was least represented in both studies (i.e., 5.5% Study 1; 10.0% Study 2). This profile represents individuals whose competence is based strongly on both self‐ and other‐referenced criteria, and whose sport/exercise activities are regulated strongly by autonomous and controlled reasons. Although research by McNeil and Wang [51] revealed a “highly motivated cluster” representing 48% of their sample, this cluster was characterized by moderate to high levels of motivational regulations and goal orientations, thus resembling both the Mixture‐Medium and Mixture‐High profiles in the current study, and with youth sport participants from an Eastern culture.

As hypothesized, the four motivational profiles we identified in both studies showed differences in risk factors for doping. More specifically, based upon the integrated motivational model [25] and past research [21], we proposed profiles exhibiting relatively high ego orientation with controlled/amotivation and low task orientation/autonomous regulation would be more at risk of doping than profiles that included high task orientation and/or high autonomous regulation. Consistent with these assertions, membership of the *Autonomous Task* profile was associated with less approving attitudes towards doping behaviors in sport and exercise contexts, with favorable doping attitudes in Study 1 being lower than for *Mixture Medium* and *Mixture High* and moral attitudes against doping being higher than for all other profiles in Study 2. This suggests that autonomous regulation, in particular, may be linked with strong moral opposition to doping at least when partnered with lower levels of controlled regulation. The elevated levels of autonomous regulation, in particular, might explain why Autonomous Task membership was associated with greater moral opposition to doping in comparison to those in the Task group. This is consistent with Barkoukis et al. [21], who found athletes with high intrinsic motivation reported significantly lower past doping use and had lower intentions to dope in the future compared to those high in extrinsic motivation or amotivation. Additionally, Autonomous Task displayed reduced risk for doping compared to most other profiles despite reporting the most frequent use of performance supplements such as creatine, caffeine, protein, and taurine. This suggests athletes in the profile appear to make a clear demarcation between the use of prohibited and non‐prohibited substances to support performance.

Similarly, those with a *Task* profile had less approving attitudes toward doping than those in the *Mixture Medium* and *Mixture High* profiles in at least one of the studies. Specifically, *Task* had less favorable attitudes toward doping than *Mixture Medium* and *Mixture High* in Study 1, weaker functional attitudes toward doping than all other profiles in Study 2, and stronger moral attitudes against doping than the *Mixture High* profile in Study 2. This profile also scored lower in moral disengagement than all other profiles in Study 2. As the Task profile had lower levels of ego orientation and controlled motivation than the other three profiles, it may be that elevated levels of one or both of these are linked with more favorable attitudes toward the functional benefits that can come from doping. Thus, the *Task* profile seems to be associated with less favorable attitudes toward doping as well as a propensity to not rationalize and justify doping. This is also consistent with Barkoukis et al. [21], who found athletes with a profile reflecting high task involvement and low ego involvement were less likely to report doping than athletes with profiles incorporating high ego involvement. Overall, our findings showed reduced risk factors for doping for profiles that included high task orientation and/or autonomous regulation when partnered with an absence of controlled regulation.

Despite the presence of high levels of task orientation and fairly high autonomous regulation within *Mixture High*, this profile showed some evidence of higher risk for doping compared to some other profiles. Specifically, in Study 1, favorable doping attitudes were higher for this profile than for any other and significantly higher than all but *Mixture Medium*. This was not really reflected in doping likelihood, though, with no significant differences between *Mixture High* and any other profile for this variable. In Study 2, *Mixture High* had higher functional attitudes supportive of doping than *Task* and lower moral attitudes opposing doping than all the other three profiles. Doping moral disengagement was also higher for *Mixture High* than for *Task*. Overall, these findings suggest that higher levels of task orientation and autonomous regulation may only offer limited protection against doping risk when ego orientation and controlled regulation are also relatively high. This finding is consistent with motivation profiling studies outside of doping. For instance, McNeil and Wang [51] compared group memberships on the purpose of sport participation among adolescent elite school players. Those exhibiting a profile reflecting moderate task orientation/high intrinsic motivation with high ego orientation and less self‐determined (controlled) motivation viewed the purpose of participation as being more for “social status” compared to those fitting a profile of high task orientation and moderate levels of more self‐determined (autonomous) motivation.

Our findings showing distinct outcomes relevant to the risk of doping for specific achievement goals depending on how athletes are regulated provide support for Vansteenkiste, Lens et al. [25] integrated model of motivation. For instance, whilst *Autonomous Task* and *Mixture High* had similar levels of task orientation, the former showed greater autonomous than controlled regulation, whereas the latter showed similar levels of autonomous and controlled regulation. Importantly, this difference was linked with differences in doping outcomes in both studies. In Study 1, *Mixture High* showed more favorable attitudes towards doping than *Autonomous Task*, and in Study 2, *Autonomous Task* had stronger moral attitudes against doping than *Mixture High*. These findings are consistent with existing studies that have linked self‐determined task‐approach goals with positive outcomes like prosocial behavior, enjoyment, satisfaction, challenge appraisals, and pride [26, 27]. Thus, motivational profiles reflecting the pursuit of task‐focused goals, high autonomous regulation, and lower controlled regulation may be linked with a range of adaptive outcomes in physically active populations.

In contrast to our findings for most other risk and protective factors for doping, we did not detect any differences in doping self‐regulatory efficacy or self‐reported doping across the four profiles in Study 2. Whilst unexpected, these two findings are consistent with one another. Whilst we were able to show differences in key risk factors for doping across motivational profiles, the low levels of doping reported for all profiles seem to indicate athletes across all four profiles felt able to control any perceived internal or external pressures to dope. Therefore, to detect differences in doping self‐regulatory efficacy, it may be necessary to recruit participants from populations in which prevalence rates for doping are known to be higher. For example, Boardley et al. [49] demonstrated the potential of such approaches when purposefully recruiting participants from hardcore bodybuilding gyms. In this study, Boardley et al. [49] reported a lifetime prevalence for prohibited performance‐enhancing drug use of 39.2% for athletes who trained in hardcore bodybuilding gyms. Adopting a similar approach in motivational profiling research would allow researchers to determine whether differences in motivational profiles reflect differences in doping self‐regulatory efficacy and self‐reported doping when internal and external pressures to dope are perceived to be higher. For self‐reported doping specifically, it is also possible that due to social desirability bias, levels of this variable were underreported to some degree (see [53]).

### 7.1 Limitations and Future Directions

Whilst these two studies have made several important and novel contributions to knowledge around motivation and doping risk, as with all research, there are some inherent limitations that need to be noted when considering the findings. First, whilst there was good consistency in the profiles identified across the two studies overall, the *Mixture Low* profile identified in Study 1 was not replicated within Study 2. It is possible that this is explained by the change in population sampled between the two studies. Study 1 sampled mostly from university sport participants, a population in which a small number of students (i.e., 5% in Study 1) whose sport participation may not be underpinned by either task or ego goals, as well as being weakly regulated by controlled motivation, autonomous motivation, or amotivation. In contrast, Study 2 specifically recruited from active gym attendees, a population in which a sub‐population of participants with low levels of any goals or motivational regulation is less likely to exist. However, it is possible that our sampling failed to capture this sub‐population, and future research should seek to confirm this profile does not exist in active gym populations. Next, we chose to measure different outcomes in Study 1 and Study 2. However, whilst this means it is not possible to make direct comparisons regarding doping risk between the two studies, we felt it was important to measure a greater range of outcomes in Study 2, and ones that provide a more complete picture of doping risk. For instance, by changing our attitude measure in Study 2, we were able to separate attitudes towards the functionality of doping from those focused on its morality. Also, by measuring self‐reported doping rather than doping likelihood in Study 2, we were able to determine whether it was possible to see differences in actual reported doping. We felt this was important because the doping likelihood measure used in Study 1 only assesses likelihood of doping in extremely specific circumstances, and ones in which many participants may never find themselves. Importantly, this measure has never been validated alongside measures of actual doping. Thus, future researchers are encouraged to validate this measure by showing scores on it are positively related to actual doping. Finally, we did not examine the potential confounds of sport type and other demographic variables (e.g., gender) on motivational profiles. However, given the largely replicated motivation profiles between the sport and exercise samples, we feel this is less of a concern. Also, our two guiding theories are considered universal theories, so from a theoretical perspective, we would not expect to see motivational profiles vary due to such demographic factors. However, future research would do well to confirm the motivational profiles identified here do not differ due to demographic factors. More generally, it would also be good to explore possible antecedents of motivational profiles to further extend motivation research conducted from a person‐centered approach.

## 8 Perspective

By conducting motivation research on doping from a person‐centered approach for the first time, this two‐study project has aided our understanding of potential causes of doping risk within sport and exercise populations, and in doing so has helped identify potential intervention targets for anti‐doping education interventions. Specifically, our findings suggested higher levels of ego goal orientation and/or controlled regulation may increase risk for doping in sport and exercise populations, even when partnered with task orientation and/or autonomous regulation. Moving forward, researchers are encouraged to collaborate with relevant stakeholders (e.g., policy makers, those designing coach development programs, and anti‐doping educators) to design, evaluate, and implement targeted interventions that seek to optimize motivational profiles and in turn reduce the risk of doping. By showing such differences across goal orientation and motivational regulation combinations, our research also provided support for Vansteenkiste, Lens et al. [25] model of motivation. Finally, the apparent replicability of these motivational profiles across sport and exercise samples suggests they endure across differing sport and exercise populations. This may be of importance beyond doping, as the differing profiles identified may also show important differences for other motivational outcomes.

## Conflicts of Interest

The authors declare no conflicts of interest.

## Supporting information


**Data S1:** sms70138‐sup‐0001‐Supinfo1.docx.

## Data Availability

The data that support the findings of this study are available on request from the corresponding author. The data are not publicly available due to privacy or ethical restrictions.

## Endnotes

^1^
Hardcore gyms are specifically focused on bodybuilding (i.e., strength training) and associated with high prevalence of androgenic anabolic steroid use and associated image and performance enhancing substances.
