# Supplementary material for: Latent Motivation Profiles and Doping in Sport and Exercise: An Integrative Approach Based on Achievement Goal and Self‐Determination Theories
Source: Scand J Med Sci Sports. 2025 Sep 19;35(9):e70138. doi: 10.1111/sms.70138 (PMC12447541; doi:10.1111/sms.70138)
Supplement: Supplementary file 1 — Data S1: sms70138‐sup‐0001‐Supinfo1.docx. [file SMS-35-e70138-s001.docx]

**Supplementary Material – Methods**

**Procedures**

**Data analysis**

Specifically, the model identification stage involved starting with specifying a single-profile model and then adding profiles to subsequent models (e.g., two-profile model, three-profile models, etc.) until ideal model solution criteria were achieved. According to Ferguson et al.’s (2020) recommendations and the common best practices for latent profile analysis, lower values for Log-Likelihood (LL), Akaike Information Criteria (AIC), Bayesian Information Criteria (BIC), and sample-size adjusted Bayesian Information Criteria (SABIC) indicated better model fit, whilst a close to 1 or higher entropy value greater than .70 indicated superior distinctiveness of the profiles in a certain latent profile model. Throughout the model development and comparison stage, we also conducted the Lo-Mendell Rubin Likelihood Ratio Test (LMRT) and the Bootstrap Likelihood Ratio Test (BLRT), examining the extent to which the subsequent model with *k* numbers of profile fits better than the prior model with *k-1* numbers of profile (see also Nylund-Gibson & Choi, 2018). Furthermore, a profile is representative and meaningful only if it contains more than 5% of the total sample (Nylund-Gibson & Choi, 2018). At the stage of model identification, one would first see decreased AIC, BIC, SABIC, increased entropy value, and significant *p*-value of the LMRT and BLRT as the number of profiles specified in a model increased until the optimal model was obtained, after which further increasing the number of profiles in the test model would lead to poorer model criteria (Ferguson et al., 2020; Nylund-Gibson & Choi, 2018). Therefore, we stopped building latent profile models for comparison when two or more of the above criteria were violated. We then retained the model prior to the last as the optimal latent profile model based on the abovementioned criteria.

With the optimal latent motivation profile model identified, we next examined the extent to which the varied motivation profiles differed in the levels of doping risk factors (e.g., doping attitude, and doping likelihood). Following recommendations for cross-profile comparison in distal outcomes (see Asparouhov & Muthén, 2021), we employed the BCH approach (Bolck et al., 2004) to test the influences of the identified motivation profiles on doping risk factors. The BCH approach is desirable because it dissolves the issue of altered profile membership when using the traditional approach (e.g., Clark & Muthén, 2009) to extend the optimal latent profile model to include additional variables considered either antecedents or outcomes of the latent profiles. The BCH approach also takes advantage of the non-parametric tests using Chi-square statistics (𝓧^2^) for cross-profile comparison, thus robust to potential data non-normality. We report mean scores and standard error (SE) of doping risk factors (e.g., doping attitude, doping likelihood) for each motivation profile, with 𝓧^2^ and the corresponding *p*-value of the comparison of each pair of profiles provided. Alpha was set at .05 level.

References

Asparouhov, T., & Muthén, B. (2021). Auxiliary Variables in Mixture Modeling: Using the BCH Method in Mplus to Estimate a Distal Outcome Model and an Arbitrary Secondary Model. https://www.statmodel.com/examples/webnotes/webnote21.pdf

Bolck, A., Croon, M., & Hagenaars, J. (2004). Estimating Latent Structure Models with Categorical Variables: One-Step Versus Three-Step Estimators. *Political Analysis, 12*(1), 3–27. <https://doi.org/10.1093/pan/mph001>

Clark, S. L., & Muthén, B. (2009). Relating latent class analysis results to variables not included in the analysis. http://hbanaszak.mjr.uw.edu.pl/TempTxt/relatinglca.pdf
